# Commissioning and initial validation of Eclipse eMC algorithm for the electron FLASH research extension (FLEX) system for pre‐clinical studies

**DOI:** 10.1002/acm2.14289

**Published:** 2024-02-06

**Authors:** Kyuhak Oh, Kyle J. Gallagher, Ying Yan, Sumin Zhou

**Affiliations:** ^1^ Department of Radiation Oncology University of Nebraska Medical Center Omaha Nebraska USA

**Keywords:** Eclipse, eFLASH, electron FLASH, eMC, FLEX, TPS commissioning, treatment planning, ultra‐high dose rate

## Abstract

**Purpose:**

To investigate the feasibility of commissioning the 16 MeV electron FLASH Extension (FLEX) in the commercial treatment planning system (TPS) for biomedical research with cell and mouse models, and in silico treatment planning studies.

**Methods:**

To commission the FLEX system with the electron Monte Carlo (eMC) algorithm in the commercial TPS, radiochromic film was used to measure the vendor‐recommended beam data. Once the beam model was generated for the eMC algorithm, supplemental measurements were collected for validation purposes and compared against the TPS‐calculated results. Additionally, the newly commissioned 16 MeV FLASH beam was compared to the corresponding 16 MeV conventional electron beam.

**Results:**

The eMC algorithm effectively modeled the FLEX system. The eMC‐calculated PDDs and profiles for the 16 MeV electron FLASH beam agreed with measured values within 1%, on average, for 6 × 6 cm^2^ and 10 × 10 cm^2^ applicators. Flatness and symmetry deviated by less than 1%, while FWHM and penumbra agreed within 1 mm for both eMC‐calculated and measured profiles. Additionally, the small field (i.e., 2‐cm diameter cutout) that was measured for validation purposes agreed with TPS‐calculated results within 1%, on average, for both the PDD and profiles. The FLASH and conventional dose rate 16 MeV electron beam were in agreement in regard to energy, but the profiles for larger field sizes began to deviate (>10 × 10 cm^2^) due to the forward‐peaked nature of the FLASH beam. For cell irradiation experiments, the measured and eMC‐calculated in‐plane and cross‐plane absolute dose profiles agreed within 1%, on average.

**Conclusions:**

The FLEX system was successfully commissioned in the commercial TPS using the eMC algorithm, which accurately modeled the forward‐peaked nature of the FLASH beam. A commissioned TPS for FLASH will be useful for pre‐clinical cell and animal studies, as well as in silico FLASH treatment planning studies for future clinical implementation.

## 1 INTRODUCTION

Ultra‐high dose rate (FLASH) radiotherapy has garnered considerable attention in recent years due to its potential to effectively kill cancer cells while minimizing harm to surrounding healthy organs and tissues. Although first introduced in the mid‐twentieth century,^1^, ^2^, ^3^ the FLASH effect was rediscovered in 2014,^4^ leading to a significant increase in research.^5^ In 2018, the first human was treated for cancer using electron FLASH,^6^ and recently, the results of the first clinical trial using proton FLASH therapy for bone metastasis were reported.^7^, ^8^

The treatment planning system (TPS) is essential for designing and delivering safe, effective, and consistent FLASH radiotherapy treatments.^9^ To date, there have been several treatment planning studies of the FLASH effect using commercial TPSs, primarily focused on proton radiotherapy.^10^, ^11^, ^12^ For electron FLASH, researchers have primarily implemented Monte Carlo codes,^13^ but few have commissioned a commercial TPS.

The purpose of this study was to investigate the feasibility of commissioning the 16 MeV electron FLASH Extension (FLEX, Varian Medical Systems, Palo Alto, CA) in the commercial treatment planning system (Eclipse v15.6, Varian Medical Systems, Palo Alto, CA) using its electron Monte Carlo (eMC) algorithm. This was accomplished by using radiochromic film to measure the vendor‐recommended beam data. We also performed an initial validation of the eMC algorithm by comparing the TPS‐calculated dose distributions to the results obtained from the radiochromic film measurements. Our study revealed that it is feasible to commission a commonly used commercial TPS with an electron FLASH (eFLASH) beam for treatment planning research and future use in patient care.

## 2 METHODS

### 2.1 FLEX system beam data collection for commissioning eMC

The FLEX system is a commercial eFLASH solution that can deliver 16 MeV electron beams at both FLASH and conventional (CONV) dose rates.^14^ Furthermore, it has been modified to achieve the ultra‐high dose rates required by FLASH radiotherapy under broad beam conditions, and the commissioning of the system has recently been published.^14^ Due to the forward‐peaked nature of the eFLASH beam produced by the FLEX system and other introduced variables (e.g., custom scattering foil), it is unknown whether the eMC algorithm in Eclipse can accurately model the novel system.

The eMC algorithm in Eclipse models particle generation, transport, and interactions to calculate dose. It has been proven to accurately model clinical high‐energy electron beams at conventional dose rates. To commission the eMC algorithm in Eclipse, the vendor requires the following measurements for each electron energy:

- A percentage depth‐dose (PDD) curve in water at 100 cm source‐to‐phantom distance (SPD) and a diagonal in‐air profile at 95 cm from the source with the collimator jaws completely open (i.e., 40 × 40 cm^2^) with no electron cone.
- A PDD curve at 100 cm SPD for each cone size with the corresponding absolute dose measured at the calibration point, for example, the depth of maximum dose (d_max_).

In‐air cross‐plane and in‐plane profiles at 95 cm from the source with the collimator jaws set to their respective programmed positions for each cone size (but without the cone) are optional for commissioning eMC. For this study, only the 6 × 6 cm^2^ and 10 × 10 cm^2^ electron applicators were commissioned due to our focus on pre‐clinical cell and mouse studies (i.e., small field sizes).

The required PDDs and profiles were all measured with radiochromic film (GafChromic EBT‐XD, Ashland Inc., Wayne, NJ, USA). Additionally, the absolute dose was measured for the required field sizes using radiochromic film at 3 cm depth (d_ma__x_) with 10 cm of water‐equivalent plastic (Solid Water HE, Sun Nuclear Corporation, Melbourne, FL, USA) for backscatter at 100 cm SPD. The radiochromic film was selected for all measurements in this study due to the ultra‐high dose rate environment with an expected uncertainty of less than 3%.^15^, ^16^ For all 16 MeV eFLASH irradiations, the delivery setting was 10 pulses with a repetition rate of 180 pulses/s. All measurements were repeated for the 16 MeV conventional dose‐rate electron beam using 600 MU/min.

### 2.2 Initial validation of eMC algorithm for eFLASH

Additional measurements were performed to validate the eMC algorithm. Radiochromic film was used to measure absolute dose and profiles for the 6 × 6 cm^2^ applicator at 1 and 3 cm depths, and for the 10 × 10 cm^2^ applicator at 1 and 3 cm depths. PDD and profiles were also measured for the 6 × 6 cm^2^ applicator with a custom 2‐cm diameter Cerrobend cutout for future small animal studies. The corresponding experimental setups were simulated in Eclipse, and the dose was calculated with the eMC algorithm. The calculated results were compared to the measured data using metrics such as symmetry, flatness, Full Width at Half Maximum (FWHM), and penumbra.

### 2.3 End‐to‐end test: dosimetric evaluation of cell experiment

**FIGURE 1:**
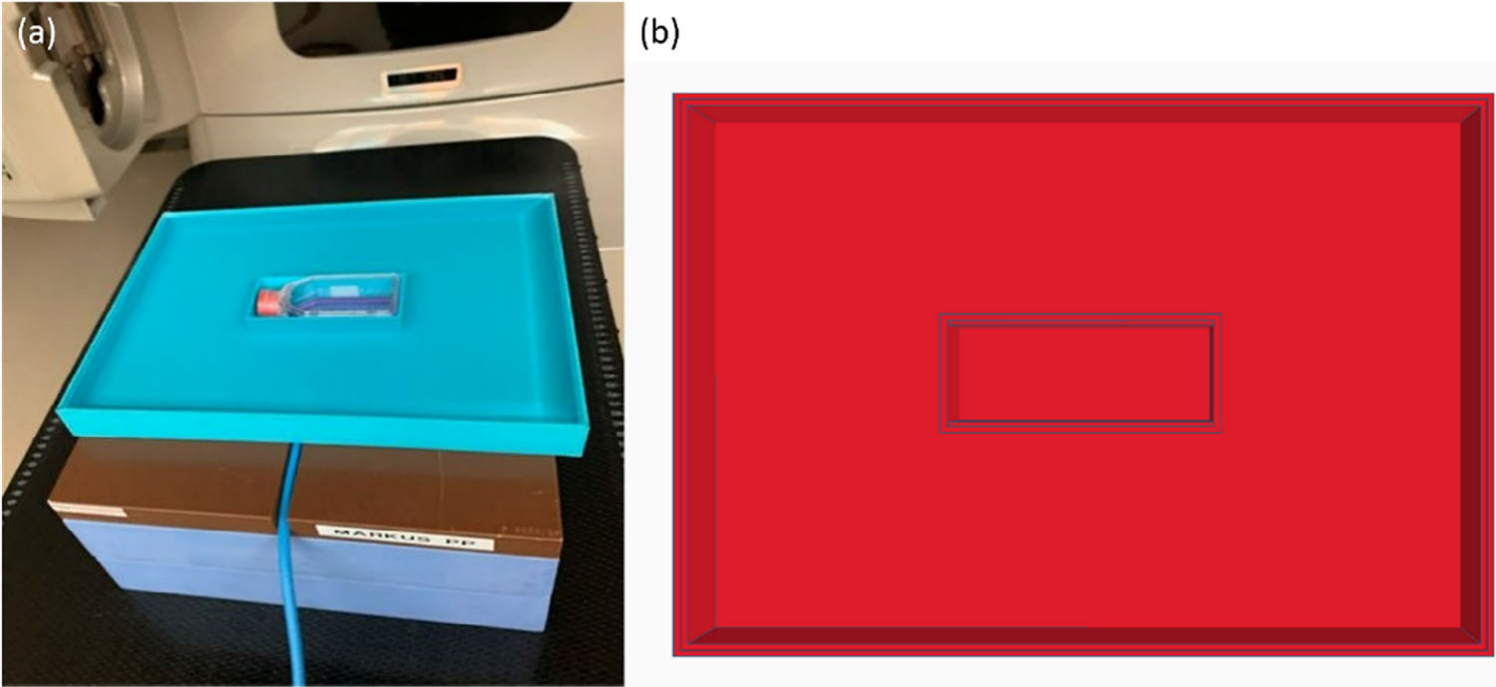
(a) Photograph of the 3D‐printed sample holder used for the cell experiments with the electron FLASH delivery system, and (b) Image of the standard tessellation language (STL) format of the sample holder.

An end‐to‐end test was conducted for cell irradiations using the FLASH environment. A customized 3D‐printed flask holder was manufactured (Figure 1). The flask containing the cell sample was located at the center of the 3D‐printed holder. The latter is a hollow structure filled with water. A total of 3 cm of water‐equivalent build‐up (1 cm of bolus and 2 cm of solid water) was placed directly on top of the 3D‐printed holder to position the cells approximately at d_max_, and 12 cm of solid water was used for adequate backscatter. The cells naturally adhered to the bottom of the flask; therefore, a radiochromic film was placed directly under the flask against the cells for in vivo dosimetry. For additional dosimetric verification, a plane‐parallel ion chamber was also positioned directly beneath the sample holder.

Human breast cancer cell lines were irradiated with a dose of 5 Gy under the following FLASH delivery conditions. The experiment employed a 16 MeV electron FLASH beam with an applicator size of 10 × 10 cm^2^, source‐to‐surface distance of 100 cm, and delivery of five pulses with a repetition rate of 180 pulses per second. To simulate this experiment in Eclipse, a computed tomography (CT) simulation image was acquired of the setup (e.g., 3D‐printed holder, bolus, solid water, and flask) and the image was imported into Eclipse for dose calculation. For the dosimetric evaluation of eMC, the absolute dose calculated by eMC was compared to the radiochromic film measurements.

## 3 RESULTS

### 3.1 FLEX system beam data collection for commissioning eMC

**FIGURE 2:**
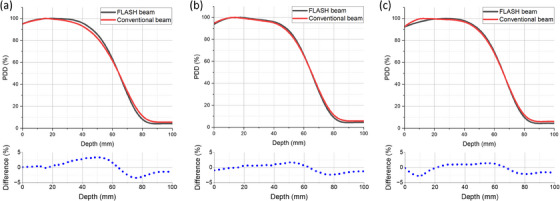
Percent depth dose (PDD) curves for both FLASH and conventional dose rate 16 MeV electron beams using (a) 6 × 6 cm^2^ applicator, (b) 10 × 10 cm^2^ applicator, (c) 40 × 40 cm^2^ open field without applicator.

Some of the following commissioning measurements were previously published^14^ and are summarized below for continuity. The measured PDDs for both the 16 MeV eFLASH and CONV beams agreed well (Figure 2), with average percentage differences of 0.43 ± 2.12%, −0.10 ± 1.26%, and −0.29 ± 1.43% for the 6 × 6 cm^2^ applicator, 10 × 10 cm^2^ applicator, and 40 × 40 cm^2^ open field without applicator, respectively. The range of 50% of the maximum dose, R_50_, values were also consistent within 0.5 mm between CONV and FLASH (Table 1). This confirmed that the FLEX system design successfully aligned the effective electron energy of the 16 MeV FLASH and CONV electron beams.

**TABLE 1:** The R_50_ of the 16 MeV eFLASH and conventional (CONV) dose‐rate electron beams for 6 × 6 cm^2^ applicator, 10 × 10 cm^2^ applicator, and 40 × 40 cm^2^ open field with no applicator.

	R_50_ (cm)	
Field size	CONV	FLASH
6 × 6 cm^2^ applicator	6.43	6.43
10 × 10 cm^2^ applicator	6.51	6.49
40 × 40 cm^2^ open field	6.61	6.62

**FIGURE 3:**
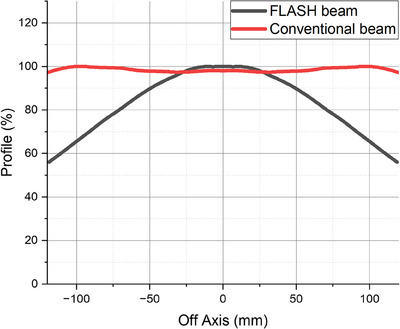
In‐air profiles for the 16 MeV eFLASH and conventional dose‐rate electron beams using a 40 × 40 cm^2^ field size (no applicator).

The in‐air profiles for both the FLASH and CONV beams using a 40 × 40 cm^2^ open field (no applicator) are shown in Figure 3. Each measurement used a single sheet of radiochromic film, which limited the measurement length of the in‐air profiles to 25.4 cm. This is longer than the diagonal length of the largest applicator size that we need for our cell and small animal studies. Therefore, this approach was satisfactory because the vendor only required the measured data of the in‐air profile to encompass the diagonal length of the largest applicator. Figure 3 demonstrates that the FLASH beam profile is forward‐peaked compared to the relatively flat CONV beam. This is because the eFLASH beam employs a thin, custom scattering foil to minimize the dose‐rate loss.

Table 2 shows the absolute dose measured at 3 cm depth (d_max_) with 100 cm SPD for the 6 × 6 cm^2^ applicator, 10 × 10 cm^2^ applicator, and the 40 × 40 cm^2^ open field (no applicator). The Monitor Unit (MU) is not applicable for the FLASH beam due to the saturation of the linac monitoring ion chambers, so the output of the FLASH beam is specified in units of Gy/pulse. Because the entry for output in Eclipse uses units of cGy/MU and has not yet implemented the concept of Gy/pulse, the FLASH beam in Eclipse still utilizes the nomenclature of MU where 1 pulse is equivalent to 100 MU. For example, to calculate the dose from one pulse delivered in reference conditions (i.e., 10 × 10 cm^2^ cone, 100 cm SSD, 3 cm depth), one would calculate the dose using a preset value of 100 MUs to attain 0.971 Gy at 3 cm depth (Table 2). Therefore, the absolute dose values in Table 2 were used for the FLASH beam commissioning of Eclipse.

**TABLE 2:** Absolute dose measurements and corresponding output factors for the 16 MeV eFLASH and conventional (CONV) dose‐rate electron beams at d_max_ for 6 × 6 cm^2^ applicator, 10 × 10 cm^2^ applicator, and 40 × 40 cm^2^ open field with no applicator. The output factors are relative to the respective 10 × 10 cm^2^ applicator.

	CONV		FLASH	
Field size	Absolute dose (cGy/MU)	Output factor	Absolute dose (Gy/pulse)	Output factor
6 × 6 cm^2^ applicator	1.076	0.988	0.973	1.002
10 × 10 cm^2^ applicator	1.089	1.000	0.971	1.000
40 × 40 cm^2^ open field	0.987	0.906	0.868	0.894

### 3.2 Initial validation of eMC algorithm for eFLASH

**FIGURE 4:**
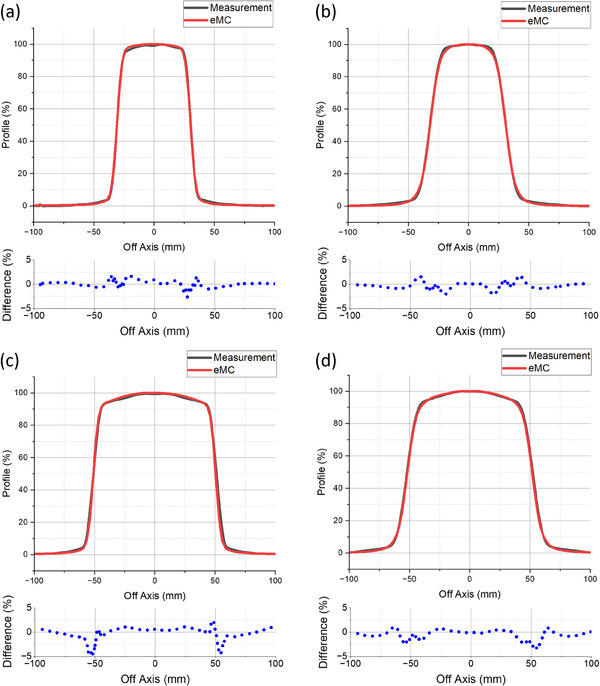
Displayed are the TPS‐computed profiles (red) compared to the respective measured profiles (black) of the eFLASH beam at depths of (a) 1 cm and (b) 3 cm for 6 × 6 cm^2^ applicator, and (c) 1 cm and (d) 3 cm for 10 × 10 cm^2^ applicator.

Figure 4 shows the comparison of the measured and eMC‐computed eFLASH profiles at various depths for the 6 × 6 cm^2^ and 10 × 10 cm^2^ applicators. The calculated and measured profiles agreed well, with average percent deviations of −0.08 ± 0.68%, 0.24 ± 0.65%, 0.31 ± 1.87%, 0.34 ± 0.85%, and 0.66 ± 0.99%, at 1 and 3 cm depths for the 6 × 6 cm^2^ applicator and at 1, 3, and 6 cm depths for the 10 × 10 cm^2^ applicator, respectively. The measured and eMC‐computed profiles were also in agreement with respect to FWHM, flatness, symmetry, and penumbra (Table 3).

**TABLE 3:** Characteristics of the measured and eMC‐computed eFLASH profiles. The FWHM, flatness, symmetry, and penumbra values for the 6 × 6 cm^2^ and 10 × 10 cm^2^ applicators were compared for the eMC‐calculated and measured eFLASH profiles.

	6 × 6 cm^2^ Applicator				10 × 10 cm^2^ Applicator			
	eMC		Measurement		eMC		Measurement	
	1 cm depth	3 cm depth	1 cm depth	3 cm depth	1 cm depth	3 cm depth	1 cm depth	3 cm depth
FWHM (cm)	6.1	6.3	6.1	6.3	10.2	10.4	10.2	10.4
Flatness (%)	3.0	9.6	3.1	9.2	3.2	5.5	3.3	4.9
Symmetry (%)	1.0	1.9	0.4	1.9	0.6	0.0	−0.2	−0.3
Penumbra (mm)	6.3	11.2	6.1	11.1	7.2	12.0	7.5	12.0

**FIGURE 5:**
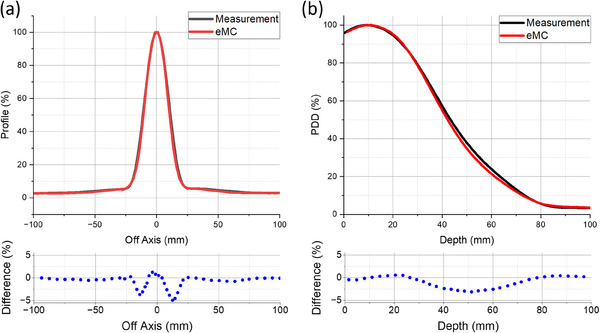
Displayed are the comparisons between the eMC‐calculated (red) and measured (black) (a) profiles at a depth of 3 cm and (b) PDDs for the 6 × 6 cm^2^ applicator with 2‐cm diameter cutout of the 16 MeV eFLASH beam.

Figure 5 illustrates the comparison of the eMC‐computed and measured eFLASH PDDs and profiles at a depth of 3 cm for a 6 × 6 cm^2^ applicator with a 2‐cm diameter circular cutout. In general, there was good agreement for which the percent deviation, on average, between the TPS calculation and measurement was −0.86 ± 1.26% and −0.58 ± 1.03% for the PDD and profile, respectively.

### 3.3 End‐to‐end test: dosimetric evaluation of cell experiment

**FIGURE 6:**
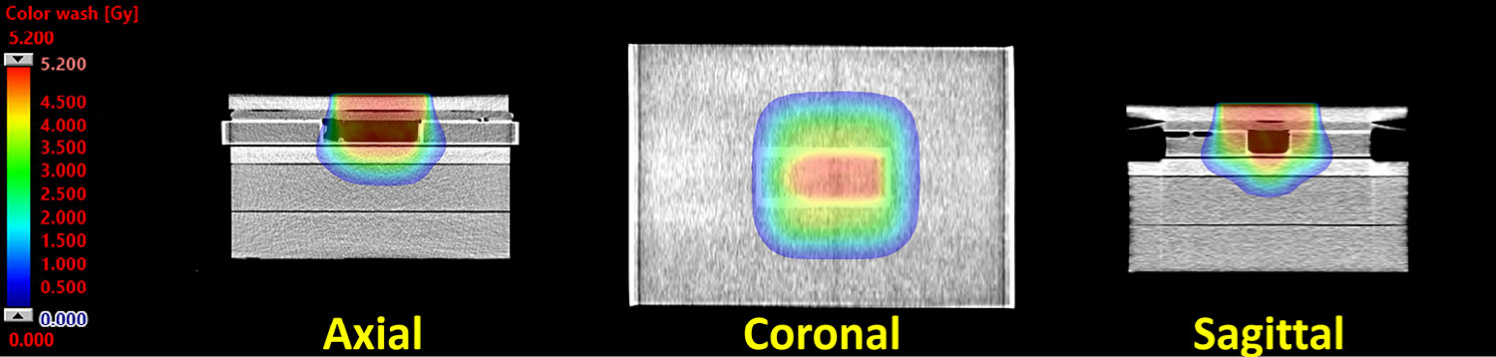
Electron FLASH dose distribution in the axial, coronal, and sagittal planes of the cell culture experiment as calculated by the eMC algorithm.

**FIGURE 7:**
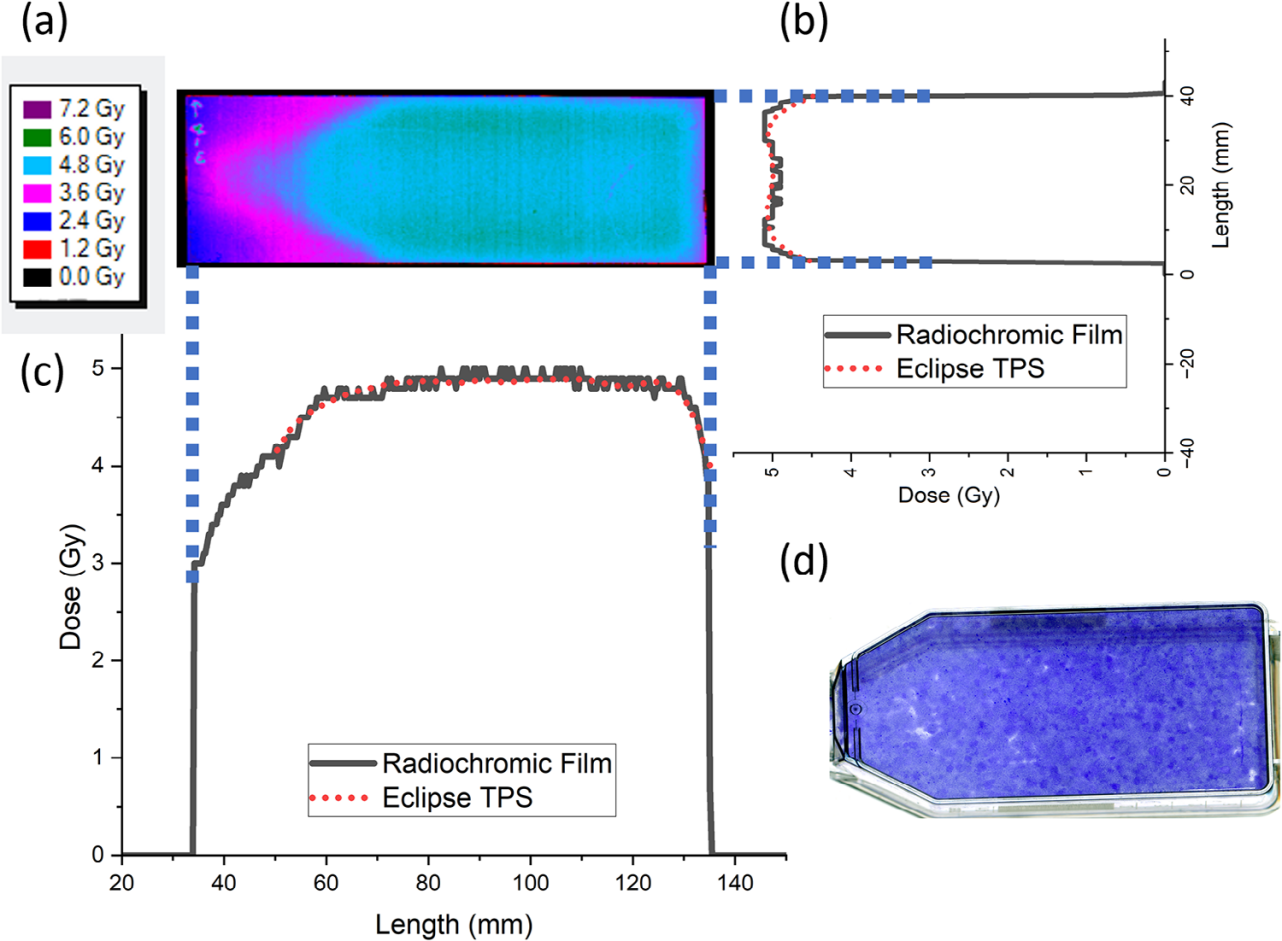
(a) 2D dose distribution measured at the bottom of the flask with radiochromic film; (b) in‐plane and (c) cross‐plane dose profiles comparing the Eclipse TPS and radiochromic film; and (d) image of the cell‐residing region within the flask (blue).

The electron FLASH dose distribution calculated by eMC for the cell experiment is displayed in Figure 6. Because the cell culture flasks were only partially filled with culture media, the additional heterogeneity of the air gap serves to further test the accuracy of the eMC algorithm. The average deviation between the radiochromic film and the eMC‐calculated in‐plane and cross‐plane dose profiles were 0.04 ± 0.10% and 0.02 ± 0.09%, respectively (Figure 7). Furthermore, the 3D‐printed sample holder was able to facilitate a relatively homogeneous dose distribution in the region where the cells resided (±4%) (Figure 7).

## 4 DISCUSSION

We successfully commissioned the Eclipse eMC algorithm for the 16 MeV eFLASH beam generated by the FLEX system. Further research is needed to evaluate the accuracy of the eMC algorithm for FLASH beams for large applicator sizes and complex irradiation conditions, but this study will be directly beneficial for future cell and small animal studies, as well as in silico FLASH treatment planning studies. Commissioning a treatment planning system is critical for future clinical trials in patients.^9^

This study had several limitations. First, the Eclipse eMC algorithm successfully calculated the physical dose distribution of the eFLASH beam but currently there is no mechanism within the TPS to view or produce a dose‐rate distribution which will be important for incorporating the FLASH effect into the treatment planning process. Second, the treatment delivery of the FLEX system is based on setting the number of pulses and not the Monitor Units as in the CONV mode.^14^ This will need to be formally incorporated into the commercial TPS for FLASH delivery. Lastly, previous research has shown that the eMC algorithm may have limitations for lower electron energies, smaller cutout sizes, and larger SSDs and may underestimate or overestimate the absorbed dose in certain regions.^17^, ^18^, ^19^, ^20^ It is important to consider these limitations when using the eMC algorithm for FLASH treatment planning, and further research is needed for geometries and conditions not tested in this study.

In conclusion, this study demonstrated the feasibility of commissioning the commercial Eclipse eMC algorithm with the novel FLEX system to accurately calculate the physical dose distributions for the 16 MeV eFLASH beam. This paves the way for future treatment planning studies and clinical trials for cancer patients who may benefit from eFLASH.

## AUTHOR CONTRIBUTIONS

Kyuhak Oh, Kyle J. Gallagher, Ying Yan, and Sumin Zhou contributed to the conception and design of the project, as well as conducting all experiments, corresponding data analysis, and writing, reviewing, and editing the manuscript. Ying Yan and Sumin Zhou (Senior author) are this work's corresponding authors.

## CONFLICTS OF INTEREST STATEMENT

The University of Nebraska Medical Center is a member of the Varian FlashForward Consortium.
